# Supplementary material for: Ecogeographic Drivers of the Spatial Spread of Highly Pathogenic Avian Influenza Outbreaks in Europe and the United States, 2016–Early 2022
Source: Int J Environ Res Public Health. 2023 Jun 1;20(11):6030. doi: 10.3390/ijerph20116030 (PMC10252585; doi:10.3390/ijerph20116030)
Supplement: Supplementary file 1 [file ijerph-20-06030-s001.zip › ijerph-2224085-supplementary.pdf]

Supplementary materials

Supplementary table S1: Downsampled dataset of 378 H5Nx HA sequences, including sequences from H5Nx outbreak in North America, December 2021- May 2022 (n=170), unpublished Massachusetts sequences from outbreak in North America acquired by our group in May 2022 (n=15), publicly available H5Nx sequences from Europe 2016-2022 (n=160), and historic sequences (n=33) resulting in a total of 380 H5 HA sequences included in the analysis.

Accession number	Source	Date	Species	Country	Continent	Location (incl masked)	Country groupings
MH819124	IRD_2.3.4.4_2014_2022	01/01/2017	Mute_swan	United_Kingdom	Europe	England	Northern_Europe
MN874967	IRD_2.3.4.4_2014_2022	01/02/2017	Duck	France	Europe	France	Central_Europe
MT027086	IRD_2.3.4.4_2014_2022	01/03/2020	Chicken	Slovakia	Europe	Slovakia	Central_Europe
MN874970	IRD_2.3.4.4_2014_2022	01/04/2017	Duck	France	Europe	France	Central_Europe
MN874975	IRD_2.3.4.4_2014_2022	01/04/2017	Chicken	France	Europe	France	Central_Europe
MT781495	IRD_2.3.4.4_2014_2022	01/04/2019	Common_buzzard	Denmark	Europe	Denmark	Northern_Europe
KY621534	IRD_2.3.4.4_2014_2022	01/06/2017	Chicken	Czech_Republic	Europe	Czech_Republic	Central_Europe
MN874981	IRD_2.3.4.4_2014_2022	01/07/2017	Chicken	France	Europe	France	Central_Europe
MN875116	IRD_2.3.4.4_2014_2022	01/10/2017	Swan	France	Europe	France	Central_Europe
MN874988	IRD_2.3.4.4_2014_2022	01/11/2017	Turkey	France	Europe	France	Central_Europe
MN874989	IRD_2.3.4.4_2014_2022	01/11/2017	Duck	France	Europe	France	Central_Europe
OM943965	IRD_2.3.4.4_2014_2022	01/11/2022	Greylag_goose	Spain	Europe	Spain	Southern_Europe
OM943964	IRD_2.3.4.4_2014_2022	01/11/2022	Grey_heron	Spain	Europe	Spain	Southern_Europe
MH819120	IRD_2.3.4.4_2014_2022	01/13/2017	Mute_swan	United_Kingdom	Europe	England	Northern_Europe
MH819127	IRD_2.3.4.4_2014_2022	01/13/2017	Mute_swan	United_Kingdom	Europe	England	Northern_Europe
MN874998	IRD_2.3.4.4_2014_2022	01/13/2017	Chicken	France	Europe	France	Central_Europe
OM943967	IRD_2.3.4.4_2014_2022	01/14/2022	Greylag_goose	Spain	Europe	Spain	Southern_Europe
MH819119	IRD_2.3.4.4_2014_2022	01/15/2017	Mute_swan	United_Kingdom	Europe	England	Northern_Europe
OM943966	IRD_2.3.4.4_2014_2022	01/15/2022	Turkey	Spain	Europe	Spain	Southern_Europe
MN875005	IRD_2.3.4.4_2014_2022	01/16/2017	Chicken	France	Europe	France	Central_Europe
MN875009	IRD_2.3.4.4_2014_2022	01/16/2017	Chicken	France	Europe	France	Central_Europe
MN874996	IRD_2.3.4.4_2014_2022	01/16/2017	Duck	France	Europe	France	Central_Europe
MN875006	IRD_2.3.4.4_2014_2022	01/17/2017	Chicken	France	Europe	France	Central_Europe
MN875007	IRD_2.3.4.4_2014_2022	01/17/2017	Guinea_fowl	France	Europe	France	Central_Europe
MN875010	IRD_2.3.4.4_2014_2022	01/17/2017	Chicken	France	Europe	France	Central_Europe
MN875004	IRD_2.3.4.4_2014_2022	01/19/2017	Common_buzzard	France	Europe	France	Central_Europe
MN875015	IRD_2.3.4.4_2014_2022	01/20/2017	Duck	France	Europe	France	Central_Europe
MN875012	IRD_2.3.4.4_2014_2022	01/21/2017	Kestelfalcon	France	Europe	France	Central_Europe
MN875014	IRD_2.3.4.4_2014_2022	01/23/2017	Duck	France	Europe	France	Central_Europe
MH819128	IRD_2.3.4.4_2014_2022	01/24/2017	Mute_swan	United_Kingdom	Europe	England	Northern_Europe
MN875017	IRD_2.3.4.4_2014_2022	01/24/2017	Chicken	France	Europe	France	Central_Europe
MN875025	IRD_2.3.4.4_2014_2022	01/24/2017	Chicken	France	Europe	France	Central_Europe
MN875018	IRD_2.3.4.4_2014_2022	01/25/2017	Duck	France	Europe	France	Central_Europe
MN875022	IRD_2.3.4.4_2014_2022	01/27/2017	Common_buzzard	France	Europe	France	Central_Europe
MN875024	IRD_2.3.4.4_2014_2022	01/27/2017	Chicken	France	Europe	France	Central_Europe

MZ166240	IRD_2.3.4.4_2014_2022	01/28/2021	Mule_duck	France	Europe	France	Central_Europe
OM943970	IRD_2.3.4.4_2014_2022	01/28/2022	White_stork	Spain	Europe	Spain	Southern_Europe
OM943968	IRD_2.3.4.4_2014_2022	01/28/2022	Cattle_egret	Spain	Europe	Spain	Southern_Europe
OM943969	IRD_2.3.4.4_2014_2022	01/28/2022	Grey_heron	Spain	Europe	Spain	Southern_Europe
MN875030	IRD_2.3.4.4_2014_2022	01/31/2017	Duck	France	Europe	France	Central_Europe
MH819118	IRD_2.3.4.4_2014_2022	01/2017	Mute_swan	United_Kingdom	Europe	England	Northern_Europe
MH819117	IRD_2.3.4.4_2014_2022	01/2017	Mute_swan	United_Kingdom	Europe	England	Northern_Europe
MF073901	IRD_2.3.4.4_2014_2022	02/01/2017	Chicken	Belgium	Europe	Belgium	Northern_Europe
MT198989	IRD_2.3.4.4_2014_2022	02/01/2017	Guinea_fowl	Belgium	Europe	Belgium	Northern_Europe
MN875035	IRD_2.3.4.4_2014_2022	02/03/2017	Duck	France	Europe	France	Central_Europe
OM943977	IRD_2.3.4.4_2014_2022	02/03/2022	White_stork	Spain	Europe	Spain	Southern_Europe
OM943976	IRD_2.3.4.4_2014_2022	02/04/2022	Greylag_goose	Spain	Europe	Spain	Southern_Europe
MW026103	IRD_2.3.4.4_2014_2022	02/06/2017	Goose	Denmark	Europe	Denmark	Northern_Europe
OM943972	IRD_2.3.4.4_2014_2022	02/06/2022	Turkey	Spain	Europe	Spain	Southern_Europe
MN875039	IRD_2.3.4.4_2014_2022	02/07/2017	Duck	France	Europe	France	Central_Europe
MF073909	IRD_2.3.4.4_2014_2022	02/08/2017	Peacock	Belgium	Europe	Belgium	Northern_Europe
MN875046	IRD_2.3.4.4_2014_2022	02/08/2017	Common_buzzard	France	Europe	France	Central_Europe
OM943974	IRD_2.3.4.4_2014_2022	02/08/2022	Turkey	Spain	Europe	Spain	Southern_Europe
OM943975	IRD_2.3.4.4_2014_2022	02/08/2022	Turkey	Spain	Europe	Spain	Southern_Europe
MT781554	IRD_2.3.4.4_2014_2022	02/09/2018	White_tailed_eagle	Denmark	Europe	Denmark	Northern_Europe
OM943973	IRD_2.3.4.4_2014_2022	02/09/2022	White_stork	Spain	Europe	Spain	Southern_Europe
OM943978	IRD_2.3.4.4_2014_2022	02/09/2022	Turkey	Spain	Europe	Spain	Southern_Europe
MN875054	IRD_2.3.4.4_2014_2022	02/10/2017	Duck	France	Europe	France	Central_Europe
MN875056	IRD_2.3.4.4_2014_2022	02/11/2017	Chicken	France	Europe	France	Central_Europe
MN875058	IRD_2.3.4.4_2014_2022	02/11/2017	Duck	France	Europe	France	Central_Europe
OM943979	IRD_2.3.4.4_2014_2022	02/11/2022	Chicken	Spain	Europe	Spain	Southern_Europe
MN875062	IRD_2.3.4.4_2014_2022	02/12/2017	Chicken	France	Europe	France	Central_Europe
MT781602	IRD_2.3.4.4_2014_2022	02/12/2018	White_tailed_eagle	Denmark	Europe	Denmark	Northern_Europe
MN875060	IRD_2.3.4.4_2014_2022	02/13/2017	Chicken	France	Europe	France	Central_Europe
MT781546	IRD_2.3.4.4_2014_2022	02/13/2018	White_tailed_eagle	Denmark	Europe	Denmark	Northern_Europe
MN875065	IRD_2.3.4.4_2014_2022	02/14/2017	Chicken	France	Europe	France	Central_Europe
OM943982	IRD_2.3.4.4_2014_2022	02/14/2022	White_stork	Spain	Europe	Spain	Southern_Europe
OM943981	IRD_2.3.4.4_2014_2022	02/14/2022	Chicken	Spain	Europe	Spain	Southern_Europe
OM943980	IRD_2.3.4.4_2014_2022	02/14/2022	Turkey	Spain	Europe	Spain	Southern_Europe
OM943983	IRD_2.3.4.4_2014_2022	02/14/2022	Chicken	Spain	Europe	Spain	Southern_Europe
MN875075	IRD_2.3.4.4_2014_2022	02/15/2017	Duck	France	Europe	France	Central_Europe
OM943986	IRD_2.3.4.4_2014_2022	02/15/2022	Turkey	Spain	Europe	Spain	Southern_Europe
OM943987	IRD_2.3.4.4_2014_2022	02/16/2022	Chicken	Spain	Europe	Spain	Southern_Europe
OM943985	IRD_2.3.4.4_2014_2022	02/17/2022	Chicken	Spain	Europe	Spain	Southern_Europe
OM943984	IRD_2.3.4.4_2014_2022	02/17/2022	Greylag_goose	Spain	Europe	Spain	Southern_Europe
MZ166316	IRD_2.3.4.4_2014_2022	02/20/2021	Mule_duck	France	Europe	France	Central_Europe
MF073917	IRD_2.3.4.4_2014_2022	02/21/2017	Mute_swan	Belgium	Europe	Belgium	Northern_Europe
MN875082	IRD_2.3.4.4_2014_2022	02/21/2017	Chicken	France	Europe	France	Central_Europe
MT272361	IRD_2.3.4.4_2014_2022	02/21/2020	Chicken	Bulgaria	Europe	Bulgaria	Southern_Europe
MN875084	IRD_2.3.4.4_2014_2022	02/24/2017	Duck	France	Europe	France	Central_Europe

MN875086	IRD_2.3.4.4_2014_2022	02/25/2017	Chicken	France	Europe	France	Central_Europe
MN875087	IRD_2.3.4.4_2014_2022	02/25/2017	Chicken	France	Europe	France	Central_Europe
MH107207	IRD_2.3.4.4_2014_2022	02/27/2017	Mallard	Belgium	Europe	Belgium	Northern_Europe
MN875091	IRD_2.3.4.4_2014_2022	02/27/2017	Duck	France	Europe	France	Central_Europe
MN875092	IRD_2.3.4.4_2014_2022	02/28/2017	Chicken	France	Europe	France	Central_Europe
MN875093	IRD_2.3.4.4_2014_2022	02/28/2017	Chicken	France	Europe	France	Central_Europe
MN875094	IRD_2.3.4.4_2014_2022	03/01/2017	Duck	France	Europe	France	Central_Europe
MK494923	IRD_2.3.4.4_2014_2022	03/02/2017	Goose	Spain	Europe	Spain	Southern_Europe
MN875096	IRD_2.3.4.4_2014_2022	03/02/2017	Chicken	France	Europe	France	Central_Europe
MW026080	IRD_2.3.4.4_2014_2022	03/03/2017	Common_buzzard	Denmark	Europe	Denmark	Northern_Europe
MN875100	IRD_2.3.4.4_2014_2022	03/04/2017	Duck	France	Europe	France	Central_Europe
MN875099	IRD_2.3.4.4_2014_2022	03/07/2017	Chicken	France	Europe	France	Central_Europe
MT272360	IRD_2.3.4.4_2014_2022	03/09/2020	Chicken	Bulgaria	Europe	Bulgaria	Southern_Europe
MT781570	IRD_2.3.4.4_2014_2022	03/14/2018	White_tailed_eagle	Denmark	Europe	Denmark	Northern_Europe
MT781562	IRD_2.3.4.4_2014_2022	03/16/2018	White_tailed_eagle	Denmark	Europe	Denmark	Northern_Europe
MN875102	IRD_2.3.4.4_2014_2022	03/18/2017	Duck	France	Europe	France	Central_Europe
MH107215	IRD_2.3.4.4_2014_2022	03/21/2017	Mute_swan	Belgium	Europe	Belgium	Northern_Europe
MN875103	IRD_2.3.4.4_2014_2022	03/21/2017	Chicken	France	Europe	France	Central_Europe
MN875105	IRD_2.3.4.4_2014_2022	03/21/2017	Duck	France	Europe	France	Central_Europe
MH107223	IRD_2.3.4.4_2014_2022	03/23/2017	Common_buzzard	Belgium	Europe	Belgium	Northern_Europe
MW026072	IRD_2.3.4.4_2014_2022	03/23/2017	Common_buzzard	Denmark	Europe	Denmark	Northern_Europe
MN875112	IRD_2.3.4.4_2014_2022	03/26/2017	Swan	France	Europe	France	Central_Europe
MT781482	IRD_2.3.4.4_2014_2022	03/26/2018	Black_headed_gull	Denmark	Europe	Denmark	Northern_Europe
MT781519	IRD_2.3.4.4_2014_2022	03/27/2018	Mute_swan	Denmark	Europe	Denmark	Northern_Europe
MT781591	IRD_2.3.4.4_2014_2022	03/27/2019	White_tailed_eagle	Denmark	Europe	Denmark	Northern_Europe
MT781586	IRD_2.3.4.4_2014_2022	04/02/2018	White_tailed_eagle	Denmark	Europe	Denmark	Northern_Europe
MT781578	IRD_2.3.4.4_2014_2022	04/03/2018	White_tailed_eagle	Denmark	Europe	Denmark	Northern_Europe
MT781490	IRD_2.3.4.4_2014_2022	04/18/2018	Common_buzzard	Denmark	Europe	Denmark	Northern_Europe
MF425637	IRD_2.3.4.4_2014_2022	06/10/2017	Chicken	Belgium	Europe	Belgium	Northern_Europe
MH107199	IRD_2.3.4.4_2014_2022	06/14/2017	Guinea_fowl	Belgium	Europe	Belgium	Northern_Europe
MF425636	IRD_2.3.4.4_2014_2022	06/15/2017	Chicken	Belgium	Europe	Belgium	Northern_Europe
MH107191	IRD_2.3.4.4_2014_2022	06/15/2017	Chicken	Belgium	Europe	Belgium	Northern_Europe
MN875115	IRD_2.3.4.4_2014_2022	06/23/2017	Swan	France	Europe	France	Central_Europe
MN875107	IRD_2.3.4.4_2014_2022	06/26/2017	Chicken	France	Europe	France	Central_Europe
MT781503	IRD_2.3.4.4_2014_2022	07/11/2018	Eider	Denmark	Europe	Denmark	Northern_Europe
MT781511	IRD_2.3.4.4_2014_2022	08/12/2018	Mute_swan	Denmark	Europe	Denmark	Northern_Europe
MT781473	IRD_2.3.4.4_2014_2022	08/27/2018	Mallard	Denmark	Europe	Denmark	Northern_Europe
MT781472	IRD_2.3.4.4_2014_2022	08/27/2018	Mallard	Denmark	Europe	Denmark	Northern_Europe
MT781527	IRD_2.3.4.4_2014_2022	08/30/2018	Pheasant	Denmark	Europe	Denmark	Northern_Europe
MT781470	IRD_2.3.4.4_2014_2022	09/03/2018	Eider	Denmark	Europe	Denmark	Northern_Europe
MT781474	IRD_2.3.4.4_2014_2022	09/03/2018	Mute_swan	Denmark	Europe	Denmark	Northern_Europe
OL638145	IRD_2.3.4.4_2014_2022	09/27/2021	Goose	Czech_Republic	Europe	Czech_Republic	Central_Europe
OL636392	IRD_2.3.4.4_2014_2022	09/27/2021	Goose	Czech_Republic	Europe	Czech_Republic	Central_Europe
MN708201	IRD_2.3.4.4_2014_2022	11/07/2016	Tufted_duck	Denmark	Europe	Denmark	Northern_Europe
MW026128	IRD_2.3.4.4_2014_2022	11/07/2016	Tufted_duck	Denmark	Europe	Denmark	Northern_Europe

MW026112	IRD_2.3.4.4_2014_2022	11/16/2016	Great_black_backed_gull	Denmark	Europe	Denmark	Northern_Europe
MW026088	IRD_2.3.4.4_2014_2022	11/16/2016	Common_gull	Denmark	Europe	Denmark	Northern_Europe
MW026120	IRD_2.3.4.4_2014_2022	11/19/2016	Mute_swan	Denmark	Europe	Denmark	Northern_Europe
MW026096	IRD_2.3.4.4_2014_2022	11/19/2017	Duck	Denmark	Europe	Denmark	Northern_Europe
MN875111	IRD_2.3.4.4_2014_2022	11/23/2017	Gull	France	Europe	France	Central_Europe
MH819856	IRD_2.3.4.4_2014_2022	11/24/2016	Mallard	Hungary	Europe	Hungary	Central_Europe
MN874912	IRD_2.3.4.4_2014_2022	12/01/2016	Duck	France	Europe	France	Central_Europe
MN874914	IRD_2.3.4.4_2014_2022	12/03/2016	Duck	France	Europe	France	Central_Europe
MN874918	IRD_2.3.4.4_2014_2022	12/06/2016	Duck	France	Europe	France	Central_Europe
MZ166252	IRD_2.3.4.4_2014_2022	12/06/2020	Mute_duck	France	Europe	France	Central_Europe
MN874921	IRD_2.3.4.4_2014_2022	12/09/2016	Duck	France	Europe	France	Central_Europe
MZ166260	IRD_2.3.4.4_2014_2022	12/10/2020	Mute_duck	France	Europe	France	Central_Europe
MN874928	IRD_2.3.4.4_2014_2022	12/12/2016	Duck	France	Europe	France	Central_Europe
MZ166276	IRD_2.3.4.4_2014_2022	12/13/2020	Mute_duck	France	Europe	France	Central_Europe
MN874937	IRD_2.3.4.4_2014_2022	12/15/2016	Duck	France	Europe	France	Central_Europe
MZ166239	IRD_2.3.4.4_2014_2022	12/15/2020	Mute_duck	France	Europe	France	Central_Europe
MN874953	IRD_2.3.4.4_2014_2022	12/16/2016	Duck	France	Europe	France	Central_Europe
MN875109	IRD_2.3.4.4_2014_2022	12/18/2016	Eurasian_wigeon	France	Europe	France	Central_Europe
MZ166284	IRD_2.3.4.4_2014_2022	12/21/2020	Mule_duck	France	Europe	France	Central_Europe
MN874943	IRD_2.3.4.4_2014_2022	12/22/2016	Duck	France	Europe	France	Central_Europe
MT781535	IRD_2.3.4.4_2014_2022	12/22/2018	White_tailed_eagle	Denmark	Europe	Denmark	Northern_Europe
OM943962	IRD_2.3.4.4_2014_2022	12/22/2021	Mute_swan	Spain	Europe	Spain	Southern_Europe
OM943963	IRD_2.3.4.4_2014_2022	12/22/2021	White_stork	Spain	Europe	Spain	Southern_Europe
MN874954	IRD_2.3.4.4_2014_2022	12/25/2016	Duck	France	Europe	France	Central_Europe
MZ166292	IRD_2.3.4.4_2014_2022	12/26/2020	Mule_duck	France	Europe	France	Central_Europe
MN874956	IRD_2.3.4.4_2014_2022	12/27/2016	Duck	France	Europe	France	Central_Europe
MZ166300	IRD_2.3.4.4_2014_2022	12/27/2020	Mule_duck	France	Europe	France	Central_Europe
MN875108	IRD_2.3.4.4_2014_2022	12/28/2016	Chicken	France	Europe	France	Central_Europe
MN874962	IRD_2.3.4.4_2014_2022	12/29/2016	Duck	France	Europe	France	Central_Europe
MH819126	IRD_2.3.4.4_2014_2022	12/30/2016	Mute_swan	United_Kingdom	Europe	England	Northern_Europe
MH819121	IRD_2.3.4.4_2014_2022	12/31/2016	Mute_swan	United_Kingdom	Europe	England	Northern_Europe
MH819123	IRD_2.3.4.4_2014_2022	12/31/2016	Mute_swan	United_Kingdom	Europe	England	Northern_Europe
MH819125	IRD_2.3.4.4_2014_2022	12/31/2016	Mute_swan	United_Kingdom	Europe	England	Northern_Europe
MH819122	IRD_2.3.4.4_2014_2022	12/31/2016	Mute_swan	United_Kingdom	Europe	England	Northern_Europe
MN874968	IRD_2.3.4.4_2014_2022	12/31/2016	Duck	France	Europe	France	Central_Europe
EU743301	IRD, Historic sequence	1979	Turkey	USA	USA	mask	mask
AB558457	IRD, Historic sequence	1981	Turkey	USA	USA	mask	mask
CY178062	IRD, Historic sequence	1982	Mallard	USA	USA	mask	mask
KP674447	IRD, Historic sequence	1985	Chicken	USA	USA	mask	mask
EU743071	IRD, Historic sequence	1987	Turkey	USA	USA	mask	mask
CY014872	IRD, Historic sequence	1988	Black_duck	USA	USA	mask	mask
CY005926	IRD, Historic sequence	1989	Herring_gull	USA	USA	mask	mask
KF435066	IRD, Historic sequence	1991	Finch	USA	USA	mask	mask
EU182309	IRD, Historic sequence	1992	Turkey	USA	USA	mask	mask
EU743019	IRD, Historic sequence	1993	Chicken	USA	USA	mask	mask

AF194990	IRD, Historic sequence	1997	Chicken	Italy	Europe	mask	mask
CY139689	IRD, Historic sequence	2000	Mallard	USA	USA	mask	mask
MH597212	IRD, Historic sequence	2003	Ruddy_turnstone	USA	USA	mask	mask
GU050008	IRD, Historic sequence	2006	Duck	USA	USA	mask	mask
KF183616	IRD, Historic sequence	2007	Mallard	Finland	Finland	mask	mask
MF145713	IRD, Historic sequence	2008	Mallard	Netherlands	Europe	mask	mask
CY047046	IRD, Historic sequence	2009	Duck	France	Europe	mask	mask
MF146146	IRD, Historic sequence	2010	White_fronted_goose	Netherlands	Netherlands	mask	mask
CY167192	IRD, Historic sequence	2012	American_green_winged_teal	USA	USA	mask	mask
CY176965	IRD, Historic sequence	2013	Mallard	USA	USA	mask	mask
KP739405	IRD, Historic sequence	2014	Chicken	USA	USA	mask	mask
GQ247849	IRD, Historic sequence	1980	Turkey	Italy	Europe	mask	mask
M18451	IRD, Historic sequence	1983	Turkey	Ireland	Europe	mask	mask
KM186131	IRD, Historic sequence	1984	Duck	USA	North_America	mask	mask
EU743233	IRD, Historic sequence	1986	Goose	USA	North_America	mask	mask
L46586	IRD, Historic sequence	1994	Chicken	Mexico	North_America	mask	mask
AB558474	IRD, Historic sequence	1995	Chicken	Mexico	North_America	mask	mask
OK205571	IRD, Historic sequence	1998	Chicken	Guatemala	North_America	mask	mask
EF597269	IRD, Historic sequence	2002	Mallard	Italy	Europe	mask	mask
MH613314	IRD, Historic sequence	2004	Laughing_gull	USA	USA	mask	mask
CY149348	IRD, Historic sequence	2011	Great_black_backed_gull	Iceland	Europe	mask	mask
OK205827	IRD, Historic sequence	1996	Chicken	Mexico	North_America	mask	mask
MZ019501	IRD, Historic sequence	1999	Chicken	Belgium	Europe	mask	mask
EPI_ISL_9869760	GISAID_170_NA_Outbreak	12/30/2021	American_wigeon	USA	USA	South_Carolina	Midwest & Mid-Atlantic
EPI_ISL_9876777	GISAID_170_NA_Outbreak	12/30/2021	American_blue_winged_teal	USA	USA	South_Carolina	Midwest & Mid-Atlantic
EPI_ISL_9880015	GISAID_170_NA_Outbreak	01/08/2022	American_wigeon	USA	USA	North_Carolina	Midwest & Mid-Atlantic
EPI_ISL_9880019	GISAID_170_NA_Outbreak	01/08/2022	Mallard	USA	USA	North_Carolina	Midwest & Mid-Atlantic
EPI_ISL_9880021	GISAID_170_NA_Outbreak	01/08/2022	Northern_pintail	USA	USA	North_Carolina	Midwest & Mid-Atlantic
EPI_ISL_9880151	GISAID_170_NA_Outbreak	01/08/2022	Gadwall	USA	USA	North_Carolina	Midwest & Mid-Atlantic
EPI_ISL_9880152	GISAID_170_NA_Outbreak	01/08/2022	Northern_shoveler	USA	USA	North_Carolina	Midwest & Mid-Atlantic
EPI_ISL_9880153	GISAID_170_NA_Outbreak	01/08/2022	American_wigeon	USA	USA	North_Carolina	Midwest & Mid-Atlantic
EPI_ISL_9909371	GISAID_170_NA_Outbreak	02/07/2022	Turkey	USA	USA	Indiana	Midwest & Mid-Atlantic
EPI_ISL_11628212	GISAID_170_NA_Outbreak	02/11/2022	Chicken	USA	USA	Virginia	Midwest & Mid-Atlantic
EPI_ISL_11628213	GISAID_170_NA_Outbreak	02/11/2022	Chicken	USA	USA	Kentucky	Midwest & Mid-Atlantic
EPI_ISL_11628214	GISAID_170_NA_Outbreak	02/11/2022	Chicken	USA	USA	Kentucky	Midwest & Mid-Atlantic
EPI_ISL_11628215	GISAID_170_NA_Outbreak	02/11/2022	Chicken	USA	USA	Kentucky	Midwest & Mid-Atlantic
EPI_ISL_11628216	GISAID_170_NA_Outbreak	12/02/2022	Turkey	USA	USA	Kentucky	Midwest & Mid-Atlantic
EPI_ISL_11628217	GISAID_170_NA_Outbreak	12/02/2022	Turkey	USA	USA	Kentucky	Midwest & Mid-Atlantic
EPI_ISL_11628218	GISAID_170_NA_Outbreak	02/14/2022	Turkey	USA	USA	Indiana	Midwest & Mid-Atlantic
EPI_ISL_11628219	GISAID_170_NA_Outbreak	02/14/2022	Turkey	USA	USA	Indiana	Midwest & Mid-Atlantic
EPI_ISL_11628220	GISAID_170_NA_Outbreak	02/14/2022	Turkey	USA	USA	Indiana	Midwest & Mid-Atlantic
EPI_ISL_11628221	GISAID_170_NA_Outbreak	02/17/2022	Chicken	USA	USA	Maine	Northeastern_USA
EPI_ISL_11628222	GISAID_170_NA_Outbreak	02/21/2022	Chicken	USA	USA	Delaware	Northeastern_USA
EPI_ISL_11628223	GISAID_170_NA_Outbreak	02/21/2022	Chicken	USA	USA	Delaware	Northeastern_USA
EPI_ISL_11628224	GISAID_170_NA_Outbreak	02/21/2022	Chicken	USA	USA	Delaware	Northeastern_USA

EPI_ISL_11628225	GISAID_170_NA_Outbreak	02/16/2022	Turkey	USA	USA	Indiana	Midwest & Mid-Atlantic
EPI_ISL_11628226	GISAID_170_NA_Outbreak	02/16/2022	Turkey	USA	USA	Indiana	Midwest & Mid-Atlantic
EPI_ISL_11628227	GISAID_170_NA_Outbreak	02/18/2022	Turkey	USA	USA	Indiana	Midwest & Mid-Atlantic
EPI_ISL_11628228	GISAID_170_NA_Outbreak	02/18/2022	Turkey	USA	USA	Indiana	Midwest & Mid-Atlantic
EPI_ISL_11628229	GISAID_170_NA_Outbreak	02/22/2022	Turkey	USA	USA	Michigan	Upper_Midwest_USA
EPI_ISL_11628230	GISAID_170_NA_Outbreak	02/22/2022	Chicken	USA	USA	Michigan	Upper_Midwest_USA
EPI_ISL_11628231	GISAID_170_NA_Outbreak	02/22/2022	Guinea_fowl	USA	USA	Michigan	Upper_Midwest_USA
EPI_ISL_11628232	GISAID_170_NA_Outbreak	02/20/2022	Chicken	USA	USA	Maine	Northeastern_USA
EPI_ISL_11628233	GISAID_170_NA_Outbreak	02/22/2022	Pheasant	USA	USA	New_York	Northeastern_USA
EPI_ISL_11628234	GISAID_170_NA_Outbreak	02/22/2022	Pheasant	USA	USA	New_York	Northeastern_USA
EPI_ISL_11628235	GISAID_170_NA_Outbreak	02/21/2022	Turkey	USA	USA	Indiana	Midwest & Mid-Atlantic
EPI_ISL_11628236	GISAID_170_NA_Outbreak	02/21/2022	Turkey	USA	USA	Indiana	Midwest & Mid-Atlantic
EPI_ISL_11628237	GISAID_170_NA_Outbreak	02/21/2022	Turkey	USA	USA	Indiana	Midwest & Mid-Atlantic
EPI_ISL_11628238	GISAID_170_NA_Outbreak	02/28/2022	Chicken	USA	USA	Iowa	Upper_Midwest_USA
EPI_ISL_11628239	GISAID_170_NA_Outbreak	02/28/2022	Chicken	USA	USA	Connecticut	Northeastern_USA
EPI_ISL_11628240	GISAID_170_NA_Outbreak	02/28/2022	Turkey	USA	USA	Indiana	Midwest & Mid-Atlantic
EPI_ISL_11628241	GISAID_170_NA_Outbreak	02/28/2022	Turkey	USA	USA	Indiana	Midwest & Mid-Atlantic
EPI_ISL_11628242	GISAID_170_NA_Outbreak	03/01/2022	Turkey	USA	USA	Indiana	Midwest & Mid-Atlantic
EPI_ISL_11628243	GISAID_170_NA_Outbreak	03/01/2022	Turkey	USA	USA	Indiana	Midwest & Mid-Atlantic
EPI_ISL_11628244	GISAID_170_NA_Outbreak	03/02/2022	Chicken	USA	USA	Missouri	Midwest & Mid-Atlantic
EPI_ISL_11628245	GISAID_170_NA_Outbreak	03/02/2022	Chicken	USA	USA	Missouri	Midwest & Mid-Atlantic
EPI_ISL_11628246	GISAID_170_NA_Outbreak	03/03/2022	Chicken	USA	USA	Maryland	Northeastern_USA
EPI_ISL_11628247	GISAID_170_NA_Outbreak	03/03/2022	Chicken	USA	USA	Maryland	Northeastern_USA
EPI_ISL_11628248	GISAID_170_NA_Outbreak	03/03/2022	Chicken	USA	USA	Missouri	Midwest & Mid-Atlantic
EPI_ISL_11628249	GISAID_170_NA_Outbreak	03/04/2022	Turkey	USA	USA	South_Dakota	North Rockies & Plains
EPI_ISL_11628250	GISAID_170_NA_Outbreak	03/04/2022	Turkey	USA	USA	South_Dakota	North Rockies & Plains
EPI_ISL_11628251	GISAID_170_NA_Outbreak	03/06/2022	Turkey	USA	USA	Iowa	Upper_Midwest_USA
EPI_ISL_11628252	GISAID_170_NA_Outbreak	03/06/2022	Turkey	USA	USA	Iowa	Upper_Midwest_USA
EPI_ISL_11897507	GISAID_170_NA_Outbreak	03/03/2022	Chicken	USA	USA	Maryland	Northeastern_USA
EPI_ISL_11897508	GISAID_170_NA_Outbreak	03/03/2022	Chicken	USA	USA	Maryland	Northeastern_USA
EPI_ISL_11897667	GISAID_170_NA_Outbreak	03/03/2022	Chicken	USA	USA	Missouri	Midwest & Mid-Atlantic
EPI_ISL_11897668	GISAID_170_NA_Outbreak	03/04/2022	Turkey	USA	USA	South_Dakota	North Rockies & Plains
EPI_ISL_11897669	GISAID_170_NA_Outbreak	03/04/2022	Turkey	USA	USA	South_Dakota	North Rockies & Plains
EPI_ISL_11897670	GISAID_170_NA_Outbreak	03/06/2022	Turkey	USA	USA	Iowa	Upper_Midwest_USA
EPI_ISL_11897671	GISAID_170_NA_Outbreak	03/06/2022	Turkey	USA	USA	Iowa	Upper_Midwest_USA
EPI_ISL_11897672	GISAID_170_NA_Outbreak	03/07/2022	Turkey	USA	USA	Missouri	Midwest & Mid-Atlantic
EPI_ISL_11897673	GISAID_170_NA_Outbreak	03/07/2022	Chicken	USA	USA	Delaware	Northeastern_USA
EPI_ISL_11897674	GISAID_170_NA_Outbreak	03/07/2022	Chicken	USA	USA	Delaware	Northeastern_USA
EPI_ISL_11897675	GISAID_170_NA_Outbreak	03/04/2022	Chicken	USA	USA	Maryland	Northeastern_USA
EPI_ISL_11897676	GISAID_170_NA_Outbreak	03/07/2022	Chicken	USA	USA	Maryland	Northeastern_USA
EPI_ISL_11897677	GISAID_170_NA_Outbreak	03/08/2022	Turkey	USA	USA	Missouri	Midwest & Mid-Atlantic
EPI_ISL_11897678	GISAID_170_NA_Outbreak	03/08/2022	Turkey	USA	USA	Missouri	Midwest & Mid-Atlantic
EPI_ISL_11897679	GISAID_170_NA_Outbreak	03/08/2022	Chicken	USA	USA	Maryland	Northeastern_USA
EPI_ISL_11897680	GISAID_170_NA_Outbreak	03/08/2022	Chicken	USA	USA	Maryland	Northeastern_USA
EPI_ISL_11897681	GISAID_170_NA_Outbreak	03/10/2022	Chicken	USA	USA	Iowa	Upper_Midwest_USA

EPI_ISL_11897682	GISAID_170_NA_Outbreak	03/10/2022	Chicken	USA	USA	Iowa	Upper_Midwest_USA
EPI_ISL_11897683	GISAID_170_NA_Outbreak	03/09/2022	Guinea_fowl	USA	USA	Illinois	Midwest & Mid-Atlantic
EPI_ISL_11897684	GISAID_170_NA_Outbreak	03/09/2022	Chicken	USA	USA	Kansas	Midwest & Mid-Atlantic
EPI_ISL_11897685	GISAID_170_NA_Outbreak	03/09/2022	Chicken	USA	USA	Kansas	Midwest & Mid-Atlantic
EPI_ISL_11897686	GISAID_170_NA_Outbreak	03/10/2022	Turkey	USA	USA	Maine	Northeastern_USA
EPI_ISL_11897687	GISAID_170_NA_Outbreak	03/10/2022	Chicken	USA	USA	Maine	Northeastern_USA
EPI_ISL_11897688	GISAID_170_NA_Outbreak	03/11/2022	Turkey	USA	USA	South_Dakota	North Rockies & Plains
EPI_ISL_11897689	GISAID_170_NA_Outbreak	03/11/2022	Turkey	USA	USA	South_Dakota	North Rockies & Plains
EPI_ISL_11897690	GISAID_170_NA_Outbreak	03/13/2022	Chicken	USA	USA	Wisconsin	Upper_Midwest_USA
EPI_ISL_11897691	GISAID_170_NA_Outbreak	03/13/2022	Chicken	USA	USA	Wisconsin	Upper_Midwest_USA
EPI_ISL_11897692	GISAID_170_NA_Outbreak	03/11/2022	Chicken	USA	USA	Maine	Northeastern_USA
EPI_ISL_11897693	GISAID_170_NA_Outbreak	03/14/2022	Chicken	USA	USA	Missouri	Midwest & Mid-Atlantic
EPI_ISL_11897694	GISAID_170_NA_Outbreak	03/14/2022	Chicken	USA	USA	Missouri	Midwest & Mid-Atlantic
EPI_ISL_11897695	GISAID_170_NA_Outbreak	03/14/2022	Chicken	USA	USA	Nebraska	North Rockies & Plains
EPI_ISL_11897696	GISAID_170_NA_Outbreak	03/14/2022	Waterfowl	USA	USA	Nebraska	North Rockies & Plains
EPI_ISL_11897697	GISAID_170_NA_Outbreak	03/15/2022	Turkey	USA	USA	New_Hampshire	Northeastern_USA
EPI_ISL_11897698	GISAID_170_NA_Outbreak	03/16/2022	Chicken	USA	USA	Delaware	Northeastern_USA
EPI_ISL_11897699	GISAID_170_NA_Outbreak	03/16/2022	Chicken	USA	USA	Delaware	Northeastern_USA
EPI_ISL_11897700	GISAID_170_NA_Outbreak	03/15/2022	Guinea_fowl	USA	USA	Maine	Northeastern_USA
EPI_ISL_11897701	GISAID_170_NA_Outbreak	03/15/2022	Chicken	USA	USA	Maine	Northeastern_USA
EPI_ISL_11897702	GISAID_170_NA_Outbreak	03/16/2022	Duck	USA	USA	Kansas	Midwest & Mid-Atlantic
EPI_ISL_11897703	GISAID_170_NA_Outbreak	03/16/2022	Chicken	USA	USA	Kansas	Midwest & Mid-Atlantic
EPI_ISL_11897704	GISAID_170_NA_Outbreak	03/17/2022	Chicken	USA	USA	Iowa	Upper_Midwest_USA
EPI_ISL_11971464	GISAID_170_NA_Outbreak	03/17/2022	Turkey	USA	USA	South_Dakota	North Rockies & Plains
EPI_ISL_11971465	GISAID_170_NA_Outbreak	03/17/2022	Turkey	USA	USA	South_Dakota	North Rockies & Plains
EPI_ISL_11971466	GISAID_170_NA_Outbreak	03/17/2022	Turkey	USA	USA	South_Dakota	North Rockies & Plains
EPI_ISL_11971467	GISAID_170_NA_Outbreak	03/17/2022	Turkey	USA	USA	South_Dakota	North Rockies & Plains
EPI_ISL_11971468	GISAID_170_NA_Outbreak	03/16/2022	Chicken	USA	USA	Kansas	Midwest & Mid-Atlantic
EPI_ISL_11971469	GISAID_170_NA_Outbreak	03/16/2022	Chicken	USA	USA	Kansas	Midwest & Mid-Atlantic
EPI_ISL_11971470	GISAID_170_NA_Outbreak	03/18/2022	Chicken	USA	USA	Maine	Northeastern_USA
EPI_ISL_11971471	GISAID_170_NA_Outbreak	03/18/2022	Chicken	USA	USA	Maine	Northeastern_USA
EPI_ISL_11971472	GISAID_170_NA_Outbreak	03/18/2022	Turkey	USA	USA	Maine	Northeastern_USA
EPI_ISL_11971475	GISAID_170_NA_Outbreak	03/19/2022	Chicken	USA	USA	Iowa	Upper_Midwest_USA
EPI_ISL_11971476	GISAID_170_NA_Outbreak	03/19/2022	Chicken	USA	USA	Iowa	Upper_Midwest_USA
EPI_ISL_11971477	GISAID_170_NA_Outbreak	03/21/2022	Chicken	USA	USA	Nebraska	North Rockies & Plains
EPI_ISL_11971478	GISAID_170_NA_Outbreak	03/21/2022	Turkey	USA	USA	South_Dakota	North Rockies & Plains
EPI_ISL_11971479	GISAID_170_NA_Outbreak	03/21/2022	Turkey	USA	USA	South_Dakota	North Rockies & Plains
EPI_ISL_11971480	GISAID_170_NA_Outbreak	03/21/2022	Turkey	USA	USA	South_Dakota	North Rockies & Plains
EPI_ISL_11971481	GISAID_170_NA_Outbreak	03/21/2022	Turkey	USA	USA	South_Dakota	North Rockies & Plains
EPI_ISL_11971482	GISAID_170_NA_Outbreak	03/21/2022	Turkey	USA	USA	South_Dakota	North Rockies & Plains
EPI_ISL_11971483	GISAID_170_NA_Outbreak	03/22/2022	Chicken	USA	USA	Maine	Northeastern_USA
EPI_ISL_11971484	GISAID_170_NA_Outbreak	03/22/2022	Turkey	USA	USA	South_Dakota	North Rockies & Plains
EPI_ISL_11971485	GISAID_170_NA_Outbreak	03/22/2022	Turkey	USA	USA	South_Dakota	North Rockies & Plains
EPI_ISL_11971486	GISAID_170_NA_Outbreak	03/22/2022	Chicken	USA	USA	South_Dakota	North Rockies & Plains
EPI_ISL_11971487	GISAID_170_NA_Outbreak	03/22/2022	Chicken	USA	USA	South_Dakota	North Rockies & Plains

EPI_ISL_11971488	GISAID_170_NA_Outbreak	03/22/2022	Turkey	USA	USA	South_Dakota	North Rockies & Plains
EPI_ISL_11971489	GISAID_170_NA_Outbreak	03/22/2022	Turkey	USA	USA	South_Dakota	North Rockies & Plains
EPI_ISL_11971490	GISAID_170_NA_Outbreak	03/22/2022	Pheasant	USA	USA	New_York	Northeastern_USA
EPI_ISL_11971491	GISAID_170_NA_Outbreak	03/22/2022	Mallard	USA	USA	New_York	Northeastern_USA
EPI_ISL_11971492	GISAID_170_NA_Outbreak	03/23/2022	Turkey	USA	USA	Iowa	Upper_Midwest_USA
EPI_ISL_11971493	GISAID_170_NA_Outbreak	03/23/2022	Turkey	USA	USA	South_Dakota	North Rockies & Plains
EPI_ISL_11971494	GISAID_170_NA_Outbreak	03/23/2022	Turkey	USA	USA	South_Dakota	North Rockies & Plains
EPI_ISL_11971495	GISAID_170_NA_Outbreak	03/22/2022	Duck	USA	USA	Michigan	Upper_Midwest_USA
EPI_ISL_11971496	GISAID_170_NA_Outbreak	03/22/2022	Goose	USA	USA	Michigan	Upper_Midwest_USA
EPI_ISL_11971497	GISAID_170_NA_Outbreak	03/22/2022	Chicken	USA	USA	Michigan	Upper_Midwest_USA
EPI_ISL_11971498	GISAID_170_NA_Outbreak	03/22/2022	Turkey	USA	USA	Michigan	Upper_Midwest_USA
EPI_ISL_11971499	GISAID_170_NA_Outbreak	03/23/2022	Chicken	USA	USA	Nebraska	North Rockies & Plains
EPI_ISL_11971500	GISAID_170_NA_Outbreak	03/24/2022	Turkey	USA	USA	South_Dakota	North Rockies & Plains
EPI_ISL_11971501	GISAID_170_NA_Outbreak	03/24/2022	Turkey	USA	USA	South_Dakota	North Rockies & Plains
EPI_ISL_11971502	GISAID_170_NA_Outbreak	03/25/2022	Pheasant	USA	USA	New_York	Northeastern_USA
EPI_ISL_12567390	GISAID_170_NA_Outbreak	03/17/2022	Chicken	USA	USA	Maryland	Northeastern_USA
EPI_ISL_12567391	GISAID_170_NA_Outbreak	03/25/2022	Chicken	USA	USA	Iowa	Upper_Midwest_USA
EPI_ISL_12567392	GISAID_170_NA_Outbreak	03/25/2022	Chicken	USA	USA	Iowa	Upper_Midwest_USA
EPI_ISL_12567393	GISAID_170_NA_Outbreak	03/25/2022	Chicken	USA	USA	Minnesota	Upper_Midwest_USA
EPI_ISL_12567529	GISAID_170_NA_Outbreak	03/25/2022	Embden_goose	USA	USA	Minnesota	Upper_Midwest_USA
EPI_ISL_12567530	GISAID_170_NA_Outbreak	03/25/2022	Turkey	USA	USA	Minnesota	Upper_Midwest_USA
EPI_ISL_12567531	GISAID_170_NA_Outbreak	03/25/2022	Turkey	USA	USA	Minnesota	Upper_Midwest_USA
EPI_ISL_12567532	GISAID_170_NA_Outbreak	03/24/2022	Duck	USA	USA	Nebraska	North Rockies & Plains
EPI_ISL_12567533	GISAID_170_NA_Outbreak	03/24/2022	Chicken	USA	USA	Nebraska	North Rockies & Plains
EPI_ISL_12690318	GISAID_170_NA_Outbreak	03/25/2022	Chicken	USA	USA	Missouri	Midwest & Mid-Atlantic
EPI_ISL_12690319	GISAID_170_NA_Outbreak	03/25/2022	Duck	USA	USA	Missouri	Midwest & Mid-Atlantic
EPI_ISL_12690320	GISAID_170_NA_Outbreak	03/25/2022	Chicken	USA	USA	South_Dakota	North Rockies & Plains
EPI_ISL_12690321	GISAID_170_NA_Outbreak	03/25/2022	Guinea_fowl	USA	USA	South_Dakota	North Rockies & Plains
EPI_ISL_12690322	GISAID_170_NA_Outbreak	03/26/2022	Turkey	USA	USA	Minnesota	Upper_Midwest_USA
EPI_ISL_12690323	GISAID_170_NA_Outbreak	03/26/2022	Turkey	USA	USA	Minnesota	Upper_Midwest_USA
EPI_ISL_12690324	GISAID_170_NA_Outbreak	03/26/2022	Turkey	USA	USA	South_Dakota	North Rockies & Plains
EPI_ISL_12690325	GISAID_170_NA_Outbreak	03/26/2022	Turkey	USA	USA	South_Dakota	North Rockies & Plains
EPI_ISL_12690568	GISAID_170_NA_Outbreak	03/26/2022	Turkey	USA	USA	South_Dakota	North Rockies & Plains
EPI_ISL_12690569	GISAID_170_NA_Outbreak	03/27/2022	Turkey	USA	USA	Iowa	Upper_Midwest_USA
EPI_ISL_12690570	GISAID_170_NA_Outbreak	03/28/2022	Turkey	USA	USA	Iowa	Upper_Midwest_USA
EPI_ISL_12690571	GISAID_170_NA_Outbreak	03/28/2022	Turkey	USA	USA	Iowa	Upper_Midwest_USA
EPI_ISL_12690589	GISAID_170_NA_Outbreak	03/28/2022	Chicken	USA	USA	Iowa	Upper_Midwest_USA
EPI_ISL_12690590	GISAID_170_NA_Outbreak	03/28/2022	Chicken	USA	USA	Iowa	Upper_Midwest_USA
EPI_ISL_12690591	GISAID_170_NA_Outbreak	03/28/2022	Turkey	USA	USA	Minnesota	Upper_Midwest_USA
EPI_ISL_12690903	GISAID_170_NA_Outbreak	03/28/2022	Turkey	USA	USA	Minnesota	Upper_Midwest_USA
EPI_ISL_12690904	GISAID_170_NA_Outbreak	03/28/2022	Turkey	USA	USA	Minnesota	Upper_Midwest_USA
EPI_ISL_12690905	GISAID_170_NA_Outbreak	03/28/2022	Chicken	USA	USA	New_York	Northeastern_USA
EPI_ISL_12690906	GISAID_170_NA_Outbreak	03/28/2022	Duck	USA	USA	New_York	Northeastern_USA
EPI_ISL_12690907	GISAID_170_NA_Outbreak	03/28/2022	American_buff_goose	USA	USA	New_York	Northeastern_USA
EPI_ISL_12690908	GISAID_170_NA_Outbreak	03/28/2022	Quail	USA	USA	New_York	Northeastern_USA

EPI_ISL_12690909	GISAID_170_NA_Outbreak	03/29/2022	Chicken	USA	USA	Wyoming	North Rockies & Plains
EPI_ISL_12690910	GISAID_170_NA_Outbreak	03/27/2022	Turkey	USA	USA	South_Dakota	North Rockies & Plains
EPI_ISL_12690911	GISAID_170_NA_Outbreak	03/27/2022	Turkey	USA	USA	South_Dakota	North Rockies & Plains
EPI_ISL_12690912	GISAID_170_NA_Outbreak	03/28/2022	Turkey	USA	USA	Minnesota	Upper_Midwest_USA
EPI_ISL_12691028	GISAID_170_NA_Outbreak	03/27/2022	Turkey	USA	USA	South_Dakota	North Rockies & Plains
EPI_ISL_12691029	GISAID_170_NA_Outbreak	03/27/2022	Turkey	USA	USA	South_Dakota	North Rockies & Plains
EPI_ISL_12691030	GISAID_170_NA_Outbreak	03/28/2022	Turkey	USA	USA	South_Dakota	North Rockies & Plains
EPI_ISL_12691031	GISAID_170_NA_Outbreak	03/28/2022	Turkey	USA	USA	South_Dakota	North Rockies & Plains
EPI_ISL_12691032	GISAID_170_NA_Outbreak	03/27/2022	Turkey	USA	USA	North_Carolina	Midwest & Mid-Atlantic
EPI_ISL_12691033	GISAID_170_NA_Outbreak	03/27/2022	Turkey	USA	USA	North_Carolina	Midwest & Mid-Atlantic
EPI_ISL_12691034	GISAID_170_NA_Outbreak	03/28/2022	Goose	USA	USA	North_Dakota	North Rockies & Plains
EPI_ISL_12691035	GISAID_170_NA_Outbreak	03/28/2022	Chicken	USA	USA	North_Dakota	North Rockies & Plains
EPI_ISL_12693855	GISAID_170_NA_Outbreak	03/31/2022	Chicken	USA	USA	Iowa	Upper_Midwest_USA
EPI_ISL_16641778	Runstadler Lab, Tufts	03/03/2022	Great_horned_owl	USA	USA	Massachusetts	Northeastern_USA
EPI_ISL_16641764	Runstadler Lab, Tufts	03/01/2022	Sanderling	USA	USA	Massachusetts	Northeastern_USA
EPI_ISL_16641765	Runstadler Lab, Tufts	03/02/2022	Sanderling	USA	USA	Massachusetts	Northeastern_USA
EPI_ISL_16641766	Runstadler Lab, Tufts	03/06/2022	Sanderling	USA	USA	Massachusetts	Northeastern_USA
EPI_ISL_16641767	Runstadler Lab, Tufts	03/06/2022	Red_tailed_hawk	USA	USA	Massachusetts	Northeastern_USA
EPI_ISL_16641768	Runstadler Lab, Tufts	03/06/2022	Sanderling	USA	USA	Massachusetts	Northeastern_USA
EPI_ISL_16641769	Runstadler Lab, Tufts	03/07/2022	Sanderling	USA	USA	Massachusetts	Northeastern_USA
EPI_ISL_16641770	Runstadler Lab, Tufts	03/07/2022	Herring_gull	USA	USA	Massachusetts	Northeastern_USA
EPI_ISL_16641771	Runstadler Lab, Tufts	03/15/2022	American_crow	USA	USA	Massachusetts	Northeastern_USA
EPI_ISL_16641772	Runstadler Lab, Tufts	03/15/2022	American_crow	USA	USA	Massachusetts	Northeastern_USA
EPI_ISL_16641773	Runstadler Lab, Tufts	03/16/2022	American_crow	USA	USA	Massachusetts	Northeastern_USA
EPI_ISL_16641774	Runstadler Lab, Tufts	03/18/2022	Sanderling	USA	USA	Massachusetts	Northeastern_USA
EPI_ISL_16641775	Runstadler Lab, Tufts	03/09/2022	American_crow	USA	USA	Massachusetts	Northeastern_USA
EPI_ISL_16641776	Runstadler Lab, Tufts	03/03/2022	Sanderling	USA	USA	Massachusetts	Northeastern_USA
EPI_ISL_16641777	Runstadler Lab, Tufts	03/02/2022	Sanderling	USA	USA	Massachusetts	Northeastern_USA

Supplementary figure S1: 95% Highest Posterior Density Tree of phylogeographic transitions between geographic regions.

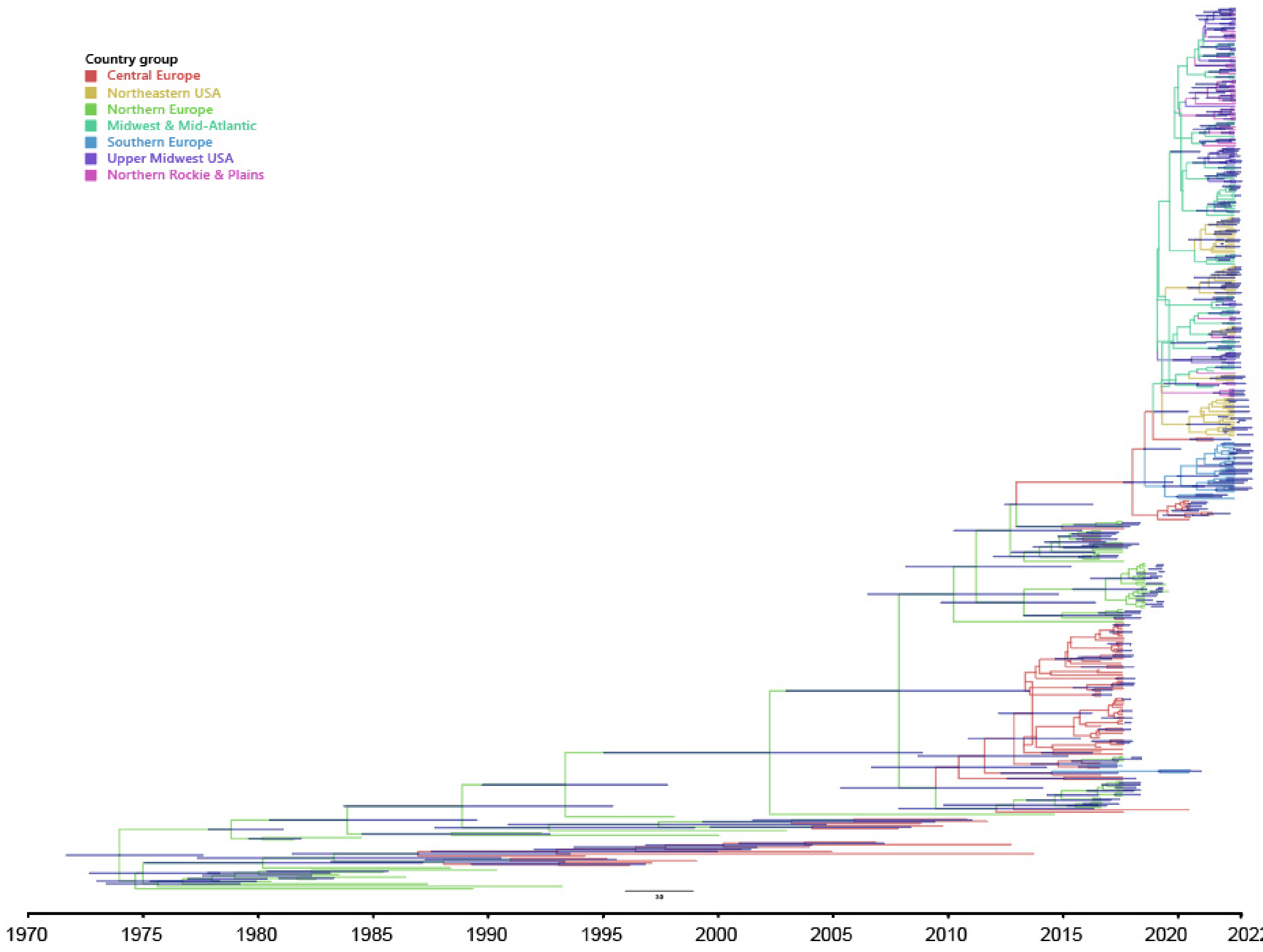

Supplementary table S2: Statistically supported transitions between geographic regions. Only BFs > 3 with corresponding posterior probability (PP) estimates > 0.5 are presented as statistically supported (noted in **bold**).

Origin	Destination	Mean transition rate (95% HPD)	Bayes Factor	Posterior probability
**Northern Europe**	**Central Europe**	**1.77 (0.49, 3.41)**	**47475.674268**	**0.9998889012**
**Central Europe**	**Southern Europe**	**0.70 (0.08, 1.54)**	**2152.9498952**	**0.9975558271**
**Midwest & Mid-Atlantic**	**Northern Rockies & Plains**	**3.19 (0.84, 5.90)**	**1526.3684523**	**0.9965559382**
**Midwest & Mid-Atlantic**	**Upper Midwest USA**	**2.88 (0.69, 5.46)**	**858.01491314**	**0.9938895678**
**Midwest & Mid-Atlantic**	**Northeast USA**	**1.18 (0.21, 1.46)**	**692.97418013**	**0.9924452839**
**Central Europe**	**Midwest & Mid-Atlantic**	**0.39 (0.01, 1.04)**	**76.307312269**	**0.9353405177**
**Central Europe**	**Northern Europe**	**0.67 (0.01, 1.86)**	**14.041828801**	**0.7269192312**
**Upper Midwest USA**	**Northern Rockies & Plains**	**1.12 (0.01, 2.94)**	**12.861269368**	**0.7091434285**
**Northern Rockies & Plains**	**Upper Midwest USA**	**1.17 (0.01, 3.13)**	**9.7362906657**	**0.6485946006**
Northern Rockies & Plains	Midwest & Mid-Atlantic	0.92 (0.00, 2.72)	4.7695515554	0.4748361293
Southern Europe	Northern Europe	0.76 (0.00, 2.47)	4.2078388707	0.4437284746
Upper Midwest USA	Midwest & Mid-Atlantic	0.91 (0.00, 2.76)	2.9824814888	0.3611820909
Northeast USA	Northern Europe	0.94 (0.00, 2.96)	1.3167547833	0.1997555827
Southern Europe	Central Europe	0.94 (0.00, 2.99)	1.2785412777	0.1950894345
Northern Rockies & Plains	Northern Europe	0.95 (0.00, 3.00)	1.1998943085	0.185312743
Upper Midwest USA	Northern Europe	0.96 (0.00, 3.07)	1.1875559978	0.1837573603
Northern Rockies & Plains	Central Europe	0.94 (0.00, 3.00)	1.1447397572	0.1783135207
Upper Midwest USA	Central Europe	0.96 (0.00, 3.16)	1.1257001343	0.1758693479
Northeast USA	Central Europe	0.94 (0.00, 2.99)	1.0430820132	0.1650927675
Southern Europe	Midwest & Mid-Atlantic	0.98 (0,00, 3.06)	0.9162080834	0.1479835574
Northeast USA	Midwest & Mid-Atlantic	0.99 (0.00, 3.15)	0.9025141189	0.1460948783
Southern Europe	Northeast USA	1.00 (0.00, 3.11)	0.7819411986	0.129096767
Northeast USA	Northern Rockies & Plains	0.98 (0.00, 3.04)	0.7603832411	0.1259860016
Southern Europe	Upper Midwest USA	0.99 (0.00, 3.08)	0.741264329	0.1232085324
Southern Europe	Northern Rockies & Plains	1.00 (0.00, 3.07)	0.741264329	0.1232085324
Northeast USA	Southern Europe	1.00 (0.00, 3.12)	0.7283325087	0.1213198533
Northeast USA	Upper Midwest USA	0.99 (0.00, 3.06)	0.7222661629	0.1204310632
Northern Rockies & Plains	Northeast USA	1.01 (0.00, 3.16)	0.6667282778	0.1122097545
Northern Rockies & Plains	Southern Europe	1.00 (0.00, 3.07)	0.6637555182	0.1117653594
Upper Midwest USA	Southern Europe	1.00 (0.00, 3.13)	0.647458366	0.1093211865

Midwest & Mid-Atlantic	Northern Europe	0.99 (0.00, 3.03)	0.6151309296	0.1044328408
Upper Midwest USA	Northeast USA	1.01 (0.00, 3.11)	0.5773778538	0.0986557049
Midwest & Mid-Atlantic	Central Europe	1.01 (0.00, 3.14)	0.5159909849	0.089101211
Central Europe	Northeast USA	0.00 (0.00, 0.00)	0.4572623194	0.0797689146
Northern Europe	Southern Europe	1.03 (0.00, 3.08)	0.3706978926	0.0656593712
Midwest & Mid-Atlantic	Southern Europe	1.02 (0.00, 3.10)	0.3619842459	0.0642150872
Central Europe	Northern Rockies & Plains	1.04 (0.00, 3.10)	0.3036995312	0.0544383957
Central Europe	Upper Midwest USA	1.03 (0.00, 3.15)	0.2795518178	0.0503277414
Northern Europe	Northern Rockies & Plains	1.06 (0.00, 3.19)	0.2691741872	0.0485501611
Northern Europe	Midwest & Mid-Atlantic	1.05 (0.00, 3.18)	0.2678797093	0.0483279636
Northern Europe	Northeast USA	1.06 (0.00, 3.20)	0.2530365428	0.0457726919
Northern Europe	Upper Midwest USA	0.99 (0.00, 3.06)	0.2478923191	0.0448839018

Supplementary Table S3: Markov rewards, by region. The mean proportion of time viruses spend within each global region between 2016-2022.

**Region**	**Mean Markov Reward**	**% of total**
Central Europe	225.57	30.0%
Northeast USA	53.08	7.1%
Northern Europe	246.46	32.8%
Midwest & Mid-Atlantic	66.71	8.9%
Southern Europe	76.63	10.2%
Upper Midwest USA	38.25	5.1%
Northern Rockies & Plains	44.82	6.0%
Total	751.52	100

Supplementary table S4. Predictors of H5Nx clade 2.3.4.4 virus diffusion within Europe and North America, 2016-2022. Variables with Bayes factors (BF) ≥ 3.0 and posterior probabilities ≥ 0.25, demonstrating statistical support, are in bold. HPD = Highest Posterior Density.

Variable name	Median coefficient	Lower 95% HPD	Upper 95% HPD	Posterior probability	Bayes factor
Latitude destination	1.214124377	-0.585555202	2.407489937	0.02629737	0.687929993
Northward movement	-1.829646159	-4.451835532	0.119607249	0.075192481	2.071005859
Precipitation origin	-0.956364177	-2.64111988	2.33769531	0.079792021	2.208674595
Precipitation change origin	-0.897593217	-2.787697887	2.197879486	0.166283372	5.080291414
Continent location USA destination	2.282530936	0.5945233	4.056957599	0.240375962	8.06028881
**Distance btw centroids**	-2.130355743	-3.277900785	-1.175432154	0.908609139	**253.2401714**
**Latitude origin**	-2.320001931	-3.767149297	-0.84596672	0.325567443	**12.29591093**
Precipitation change destination	-0.678979138	-1.115803299	-0.222710759	0.161683832	4.912663491
Precipitation destination	-0.589918783	-1.268639864	0.112277783	0.050894911	1.365898158
Samples origin	0.282552543	-1.541599376	1.887745797	0.00939906	0.241681806
Continent location Europe origin	-2.001425813	-4.076301642	-0.345651198	0.115588441	3.32903425
**Continent location Europe destination**	-2.27184237	-3.953716411	-0.579292985	0.270272973	**9.434099517**
Samples destination	0.426434773	0.02754633	1.538648832	0.00639936	0.164052537
Shared borders	2.758213248	-0.726980255	4.79373512	0.113288671	3.25433676
Temperature origin	-0.331079252	-3.695629007	3.667588749	0.123287671	3.581960579
**Temperature change origin**	-2.185978944	-3.689385204	-0.551772667	0.406459354	**17.44315035**
**Temperature change destination**	-1.248416065	-1.904850985	-0.564619541	0.267073293	**9.281713948**
Temperature destination	-0.936217257	-1.88072551	0.254658779	0.03139686	0.825654999
**Continent location USA origin**	0.928503881	0.225287933	1.673362863	0.8615	**6.220216606**
